# Supplementary material for: Male ant reproductive investment in a seasonal wet tropical forest: Consequences of future climate change
Source: PLoS One. 2022 Mar 31;17(3):e0266222. doi: 10.1371/journal.pone.0266222 (PMC8970379; doi:10.1371/journal.pone.0266222)
Supplement: S1 File — (DOCX) [file pone.0266222.s004.docx]

**S2. Analysis of stationarity and Postestimation tests**

**S2.1 Analysis of stationarity.** Before estimating the model, it is important to analyze the stationarity of the process. We do this with the Augmented Dickey-Fuller unit root test, asking whether a time series variable is stationary or non-stationary. In the case of non-stationary series, they must be differenced to make them stationary.

**Table S2.1.** Results of the Augmented Dickey-Fuller unit root Test. Significant Variables in Table 1 are scored either Yes/No for Stationarity and Differentiation.

|  |  |  |
| --- | --- | --- |
| **Variable** | **Stationarity** | **Differenced** |
| Average Abundance_7d | No | Yes |
| Accum_humidity_1y | No | Yes |
| Min_temperature_7d | No | Yes |
| Accum_humidity_1y (3m lag) | No | Yes |
| Days_rain_7d (Dry Season) | No | Yes |
| Decr_rain_2w | No | Yes |
| Ratio_litterfall_7d/2w | No | Yes |

**S2.2. Postestimation tests.** Postestimation tests examine relations between variables and residual’s structure. Testing for the effects of the latter is critical because the presence of serial correlation and for the white noise structure suggests that the model is not adequate.

**Table S2.2.1 Multicollinearity.** Multicollinearity occurs when two or more predictors in the model are correlated, producing redundant information related to the dependent variable. One way to detect the presence of multicollinearity is through variance inflation factors (VIF). In general, VIF values exceeding 4.0 denote multicollinearity, but it is considered acceptable to work with values less than 10 (Hair et al., 2010).

| **Variable** | **VIF** |
| --- | --- |
| Accum_humidity_1y | 4.289 |
| Min_temperature_7d | 1.773 |
| Accum_humidity_1y (3m lag) | 6.606 |
| Days_rain_7d (Dry Season) | 1.644 |
| Decr_rain_2w | 1.314 |
| Ratio_litterfall_7d/2w | 2.632 |
| Average Abundance_7d | 3.386 |
| Average Abundance_2w | 3.545 |
| Abundance_7w | 1.198 |
| Model moving Average (5) | 1.251 |

**Table S2.2.2 Stability conditions.** Furthermore, the model will be dynamically stable if the roots of the characteristic equation are inside the unit circle. All the values in the following table satisfy this condition; therefore, the model is stable.

|  |  |  |  |  |  |  |  |  |  |  |
| --- | --- | --- | --- | --- | --- | --- | --- | --- | --- | --- |
| **Associated Eigenvalue** | | | | | | | | | | |
| **Variable** | **1** | **2** | **3** | **4** | **5** | **6** | **7** | **8** | **9** | **10** |
| Accum_humidity_1y | 0.009 | 0.903 | 0.022 | 0.043 | 0.006 | 0.014 | <0.001 | <0.001 | <0.001 | <0.001 |
| Min_temperature_7d | 0.197 | 0.055 | 0.005 | 0.361 | 0.380 | <0.001 | <0.001 | <0.001 | <0.001 | <0.001 |
| Accum_humidity_1y (3m lag) | 0.287 | 0.611 | 0.005 | 0.003 | 0.000 | 0.092 | <0.001 | <0.001 | <0.001 | <0.001 |
| Days_rain_7d (Dry Season) | 0.058 | 0.326 | 0.240 | 0.195 | 0.178 | 0.001 | <0.001 | <0.001 | <0.001 | <0.001 |
| Decr_rain_2w | 0.065 | 0.000 | 0.878 | 0.038 | 0.016 | <0.001 | <0.001 | <0.001 | <0.001 | <0.001 |
| Ratio_litterfall_7d/2w | 1.000 | <0.001 | <0.001 | <0.001 | <0.001 | <0.001 | <0.001 | <0.001 | <0.001 | <0.001 |
| Average Abundance_7d | 0.001 | 0.003 | 0.000 | 0.004 | 0.004 | 0.000 | 0.901 | 0.007 | 0.080 | <0.001 |
| Average Abundance_2w | 0.002 | <0.001 | 0.017 | 0.043 | 0.043 | 0.005 | 0.854 | 0.000 | 0.076 | <0.001 |
| Average Abundance_7w | 0.025 | 0.022 | 0.056 | 0.023 | 0.023 | <0.001 | 0.038 | 0.820 | 0.007 | <0.001 |
| Model moving Average (5) | 0.002 | 0.020 | 0.082 | 0.040 | 0.040 | 0.011 | 0.020 | 0.003 | 0.026 | 0.789 |

**Table S2.2.3 White-noise residuals.** We used a correlogram to interpret a set of autocorrelation coefficients and test the hypothesis that the residuals are white noise. First, we visually inspected plots of the autocorrelation (ACF) and partial autocorrelation (PACF) functions of the time series. In this case, if all values of ACF and PACF are statistically null, there is evidence of white-noise residuals. Second, we used the Q statistic. Both alternatives lead to the same conclusion.


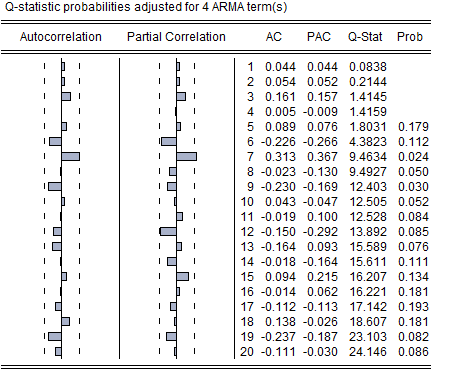


**Table S2.2.4 Normality of residuals.** We used a graph and the Jarque–Bera test to check for normality. This test allows analyzing if the skewness and kurtosis of the residuals match a normal distribution. The null hypothesis corresponds to the data is normally distributed. According to visual inspection of Figure A2.5 and by analyzing the results of Table A2.5 (p-value = 0.565), we conclude that the residuals matched a normal distribution.

| **Variable Score** | |
| --- | --- |
| Mean | -0.396 |
| Median | -2.290 |
| Maximum | 24.182 |
| Minimum | -23.898 |
| Std. Dev. | 11.787 |
| Skewness | 0.368 |
| Kurtosis | 2.646 |
| Jarque-Bera | 1.140 |
| Probability | 0.565 |

Residuals


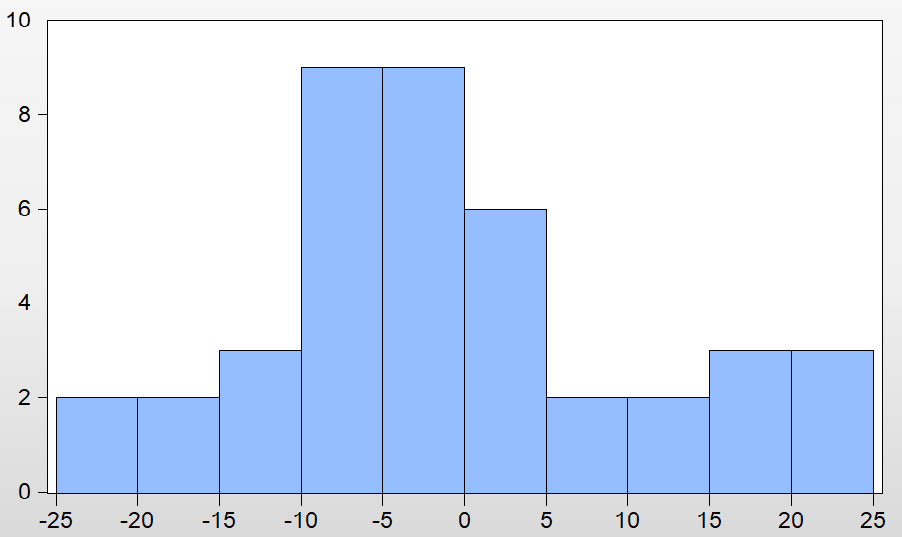


**Table S2.2.5 Serial Correlation.** The Breusch–Godfrey serial correlation LM test is a test for examining autocorrelation in the errors in a regression model. The null hypothesis is that there is no serial correlation. Because of the results obtained in Table A2.2.5, we conclude no serial correlation in the proposed model.

| **Variable** | **Coefficient** | **Std. Error** | **t** | **p.** |
| --- | --- | --- | --- | --- |
| Accum_humidity_1y | -0.04 | 1.756 | -0.002 | 1 |
| Min_temperature_7d | 0.277 | 1.193 | 0.232 | 0.82 |
| Accum_humidity_1y (3m lag) | 0.074 | 1.299 | 0.057 | 0.95 |
| Days_rain_7d (Dry Season) | 0.130 | 1.421 | 0.091 | 0.93 |
| Decr_rain_2w | -0.054 | 1.797 | -0.030 | 0.98 |
| Ratio_litterfall_7d/2w | -13.890 | 78.969 | -0.175 | 0.86 |
| Average Abundance_7d | -0.380 | 0.441 | -0.861 | 0.40 |
| Average Abundance_2w | -0.254 | 0.339 | 0.751 | 0.46 |
| Average Abundance_7w | 0.048 | 0.115 | 0.419 | 0.68 |
| Model moving Average (5) | -0.008 | 0.033 | -0.269 | 0.79 |
| RESID(-1) | 0.417 | 0.471 | 0.885 | 0.38 |
| RESID(-2) | 0.226 | 0.281 | 0.803 | 0.43 |
| R-squared | 0.029 |  |  |  |
|  |  |  |  |  |

**Table S2.2.6 Heteroskedasticity.** The Breusch–Pagan test is used to determine if the variance of the errors from regression is dependent on the values of the independent variables. In that case, the test concludes that there is heteroskedasticity in the model. The null hypothesis of homoskedasticity was not rejected in our model.

| **Variable** | **Coefficient** | **Std. Error** | **t-Statistic** | **Prob.** |
| --- | --- | --- | --- | --- |
| Accum_humidity_1y | -11.664 | 583.531 | -0.025 | 1 |
| Min_temperature_7d | 8.357 | 40.698 | -0.286 | 0.78 |
| Accum_humidity_1y (3m lag) | 9.792 | 17.937 | 0.465 | 0.64 |
| Days_rain_7d (Dry Season) | 19.437 | 30.477 | 0.321 | 0.75 |
| Decr_rain_2w | -2.970 | 13.710 | 1.417 | 0.17 |
| Ratio_litterfall_7d/2w | -269.566 | 39.667 | -0.074 | 0.94 |
| Constant | -14.821 | 489.030 | -0.551 | 0.59 |
| R-squared | 0.072 |  |  |  |
| *Dependent variable Resid^2^ | |  |  |  |
|  |  |  |  |  |


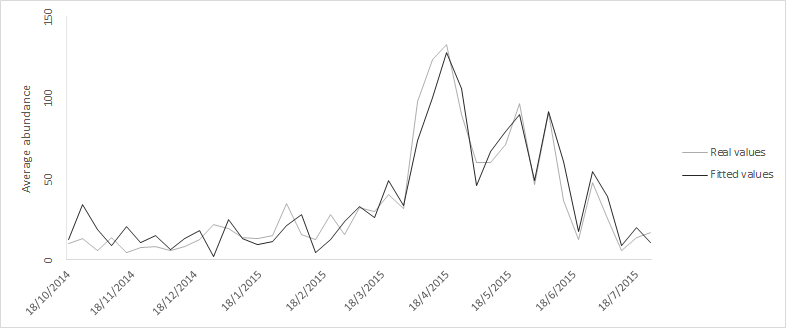


**Figure S2.2.7** **Validation test.** Additionally, for testing the adequacy of the model, authors as Enders (2015) recommend estimating a new model using only a portion of the data set and then forecasting the known values of the variable. This practice makes it possible to compare actual values with predicted values, and it is also a way of evaluating the adequacy of the model. The figure shows the actual values of average abundance vs. the number of consecutive days of rain in dry weather in the previous one week of collection. It is thus possible to conclude that our model is adequate.
