# Supplementary material for: Male ant reproductive investment in a seasonal wet tropical forest: Consequences of future climate change
Source: PLoS One. 2022 Mar 31;17(3):e0266222. doi: 10.1371/journal.pone.0266222 (PMC8970379; doi:10.1371/journal.pone.0266222)
Supplement: S2 File — (DOCX) [file pone.0266222.s005.docx]

**S5 File. Relationship between male size and phenology variables**

**Figure A5.1 Male size vs. mean angle.** Three measures of male size (Wing Width, Wing Length, and Weber’s Length) against Mean Angle. To perform this analysis, we re-scaled the mean angle for it to start not on January 1^st^, but on November 22^nd^, when the dry season starts. Rescaling the mean angle implies that ants flying close to 0 degrees fly early in the dry season, and ants flying close to 360 degrees fly late in the wet season. Mean Angle explained 11.3% of Wing Width variability (p < 0.001); 9.4% of Wing Length variability (p = 0.002); and 8.6% of Weber Length variability (p = 0.003).

**Figure A5.2. Male size vs. seasonality.** Boxplot with data of center and variance of male’s size (Wing Length, Wing Width, Weber Length), in relation to the seasonality. No significant differences were found. Wing Length (F = 0.876; p = 0.352), Wing Width (F = 1.744 p = 0.19), Weber Length (F = 0.782 p = 0.379).

**Table A5.1. Subfamily vs. mean angle.** Analysis of variance (ANOVA) between ant subfamily with Mean Angle.

|  | **Subfamilies** | |
| --- | --- | --- |
|  | F | p-value |
| **Mean Angle** | 0.688 | 0.563 |
